# Single-molecule studies on the mechanical interplay between DNA supercoiling and H-NS DNA architectural properties

**DOI:** 10.1093/nar/gku566

**Published:** 2014-07-18

**Authors:** Ci Ji Lim, Linda J. Kenney, Jie Yan

**Affiliations:** 1 Graduate School for Integrative Sciences and Engineering, National University of Singapore, Singapore; 2 Centre for Bioimaging Sciences, National University of Singapore, Singapore; 3 Mechanobiology Institute, Singapore; 4 Department of Physics, National University of Singapore, Singapore; 5 Jesse Brown Veterans Affairs Medical Center, Chicago, IL, USA; 6 Department of Microbiology & Immunology, University of Illinois-Chicago, Chicago, IL, USA; 7 Department of Biological Sciences, National University of Singapore, Singapore

## Abstract

The *Escherichia coli* H-NS protein is a major nucleoid-associated protein that is involved in chromosomal DNA packaging and gene regulatory functions. These biological processes are intimately related to the DNA supercoiling state and thus suggest a direct relationship between H-NS binding and DNA supercoiling. Here, we show that H-NS, which has two distinct DNA-binding modes, is able to differentially regulate DNA supercoiling. H-NS DNA-stiffening mode caused by nucleoprotein filament formation is able to suppress DNA plectoneme formation during DNA supercoiling. In contrast, when H-NS is in its DNA-bridging mode, it is able to promote DNA plectoneme formation during DNA supercoiling. In addition, the DNA-bridging mode is able to block twists diffusion thus trapping DNA in supercoiled domains. Overall, this work reveals the mechanical interplay between H-NS and DNA supercoiling which provides insights to H-NS organization of chromosomal DNA based on its two distinct DNA architectural properties.

## INTRODUCTION

The right-handed DNA double-helix has a helical pitch of *h* = 3.6 nm, containing 10.5 DNA base pairs (bp) with two DNA strands winding over each other once per helical turn. The number of the times the two strands wind each other is defined as the linking number and a torsionally unconstrained DNA with *N* bp and a contour length *L* would have a relaxed linking number, *Lk*_0_, calculated by *N*/10.5 or by *L/h*. For torsionally constrained DNA, winding or unwinding can cause a change in its linking number; Δ*Lk* = *Lk*-*Lk*_0_. Often, the superhelical density (also referred to as specific linking number change), *σ* = Δ*Lk/Lk*_0_, is used to describe the change in DNA linking number from its relaxed state, whereby negative or positive *σ* values refer to unwound or wound DNA, respectively. The change in linking number of a torsionally constrained DNA can then be described by DNA twists and writhes/coils as shown by the White's theorem (1,2): Δ*Lk* = *Tw* +*Wr*, where *Tw* is the twist of DNA and *Wr* is called ‘writhe’ which only depends on the bending of the double-helix DNA backbone.

*Escherichia coli* bacterium has a large circular chromosome that is (−) supercoiled (3) and its supercoiling state is tightly regulated to drive numerous important biological processes, such as DNA replication (4,5), gene expression (6–8) and chromosomal DNA organization (9,10). The process of supercoiling state homeostasis is controlled by the adenosine triphosphate-dependent topoisomerases, structural maintenance of chromosomes proteins (SMCs) and a set of abundant DNA binding architectural proteins known as the nucleoid-associated proteins (NAPs) (11). Although the mechanisms of regulating DNA supercoiling by both topoisomerases and SMCs are well characterized, those of NAPs have been less understood. This knowledge is important given that NAPs—which have approximately a dozen different species in *E. coli*—are important for chromosomal DNA packaging and global gene regulation (12,13).

In this study, we focused on an important NAP in *E. coli*, the histone-like nucleoid structuring protein (H-NS). H-NS is highly expressed during the exponential growth phase of *E. coli* and plays a critical role in chromosomal DNA organization (14–16). It also serves as a global gene silencer (14,16–18), especially for silencing laterally acquired genes (19). The negatively supercoiled *E. coli* nucleoid naturally suggests that H-NS needs to associate with supercoiled DNA to mediate some or all of its biological functions. Indeed, previous studies have demonstrated that H-NS can constrain DNA supercoiling *in vivo* and *in vitro* (14,15) but the underlying mechanism(s) was not well understood. H-NS capability in modifying DNA architecture may provide some insights.

H-NS is capable of two DNA-binding modes—a DNA-stiffening mode caused by rigid H-NS nucleoprotein filament formation (20) and a DNA bridging-mode presumably resulting from H-NS dimers (21) or mediated by the H-NS filament (22)—that is regulated by divalent cations (23). Both DNA-binding modes have been widely proposed to be important in mediating H-NS gene silencing and DNA packaging functions (24–27) but to a lesser extent in regulating DNA supercoiling (28). To address how H-NS DNA architectural properties affect DNA supercoiling, the effects of H-NS two distinct DNA-binding modes on DNA supercoiling need to be considered. The magnetic tweezers provide such a means to study DNA-protein interactions at a single-molecule level, whereby the DNA supercoiling level (also the DNA Δ*Lk*) and tension can be precisely controlled.

In the present work, using magnetic tweezers to perform single-DNA supercoiling experiments, we show that the H-NS DNA-stiffening mode suppresses formation of DNA plectonemes during DNA winding, but after DNA plectonemes are formed, the H-NS DNA-stiffening mode is able to weakly stabilize DNA in its plectoneme form. In addition, we found that the suppression of DNA plectoneme formation by H-NS DNA-stiffening mode also led to DNA melting-like behavior during DNA unwinding. In contrast, H-NS other DNA-binding mode—DNA-bridging—showed opposite effects by promoting DNA plectoneme formation during DNA winding or unwinding and can potentially block diffusion of twists across H-NS stabilized DNA plectonemes to form isolated supercoiled DNA domains. This work thus demonstrates a complex interplay between DNA supercoiling and DNA physical organization by H-NS, and potentially provides novel insights to how H-NS performs its *in vivo* functions.

## MATERIALS AND METHODS

### Protein expression and purification

The *hns* gene was cloned into pET-28 expression vector for expression in *E.coli* BL21. The expressed H-NS protein has a C-terminal 6X-HIS-tag for performing affinity-tag purification. The expression and purification protocol was essentially the same as previously described (23,26). Briefly, transformed BL21 cells were induced with Isopropyl β-D-1-thiogalactopyranoside (IPTG) and lysed by sonication. Overexpressed His-tagged H-NS was purified using nickel-column chromatography and then further concentrated by gel filtration. The proteins were dialyzed against 25 mM Tris, 500 mM KCl, pH 7.5 to remove any residual imidazole. H-NS protein was stored in −20°C in 50% glycerol. H-NS protein purity was verified by Sodium dodecyl sulphate-polyacrylamide gel electrophoresis and the concentration quantified using optical absorbance at 280 nm and converted to molarity using the calculated H-NS extinction coefficient.

### DNA construction for magnetic tweezers experiments

Nick-free pRL574 DNA (torsionally constrained) with multiple biotin and digoxigenin at each end was constructed for the supercoiling experiments. Briefly, plasmid pRL574 (29) (7474 bp) was digested using XhoI and BamHI restriction enzymes and then ligated with multiple digoxigenin- and biotin-labeled 500 bp DNA handles. The digoxigenin-labeled handles were XhoI-digested while the biotin-labeled handles were BamHI-digested. Torsionally unconstrained DNA tether can always be found in the flow-channel since it is impossible to eliminate all DNA-nicking events during sample preparation and these DNA tethers were used for torsionally unconstrained DNA experiments. λ-DNA construct containing biotin at one end and digoxigenin at the other end was made according to well-established protocols (23).

### Magnetic tweezers experiment setup

A simple micro flow-channel was constructed using two #1.5 coverslips held by melted parafilm. Typical channel volume was about 40–50 μl. Channel buffer exchange was done by adding the desired buffer at one end of the channel using Kimwipes (Kimberly-Clark) to draw from the other end. The bottom coverslip was coated with PEG-NHS (Silane-PEG-NHS, Nanocs) as previously described (30). Note that 0.1 mg/ml Anti-digoxigenin fragments (Roche) and 0.01% 3 μm amino-coated polystyrene beads were then grafted onto the surface through the NHS groups attached to the exposed ends of the surface PEG by incubating the reactants in 1X PBS (phosphate buffered saline) buffer for 1 h. The remaining unbound Anti-digoxigenin fragments and beads were washed away with 1 X PBS buffer and the channels were incubated with 1% bovine serum albumin to block any remaining uncoated glass surface before use.

A total of 0.5–1.0 ng/μl of DNA was added to the flow channel and incubated for 10 min. Note that 50 μg/ml of 1 μm sized streptavidin-coated magnetic beads (Dynabeads MyOne Streptavidin, Invitrogen) was then added and incubated for another 10 min to form DNA tethers. The unbound magnetic beads and DNA were then removed by washing with experimental buffer before performing experiments. The magnetic tweezers setup is similar to that previously described (31). Briefly, a pair of Neodymium magnets was mounted onto a micromanipulator (MP-285, Sutter Instruments) and a rotation stage (M660, Physik Instruments) for three-dimensional (X,Y,Z) control and rotation, respectively. The tethered beads positions were tracked with 5 nm spatial resolution at 80 Hz. The buffers used in the experiments were either stiffening buffer (10 mM Tris, 50 mM KCl, pH 7.5) or bridging buffer (10 mM Tris, 50 mM KCl, 10 mM MgCl_2_, pH 7.5). All experiments were conducted at 23 ± 1°C. Force-extension curve (FEC) measurements were performed by measuring DNA extension averaged for 10 s at different DNA-stretching forces. Turns-extension curve (TEC) measurements were similarly performed but at constant force and at different DNA turns.

## RESULTS

**Figure 1.**
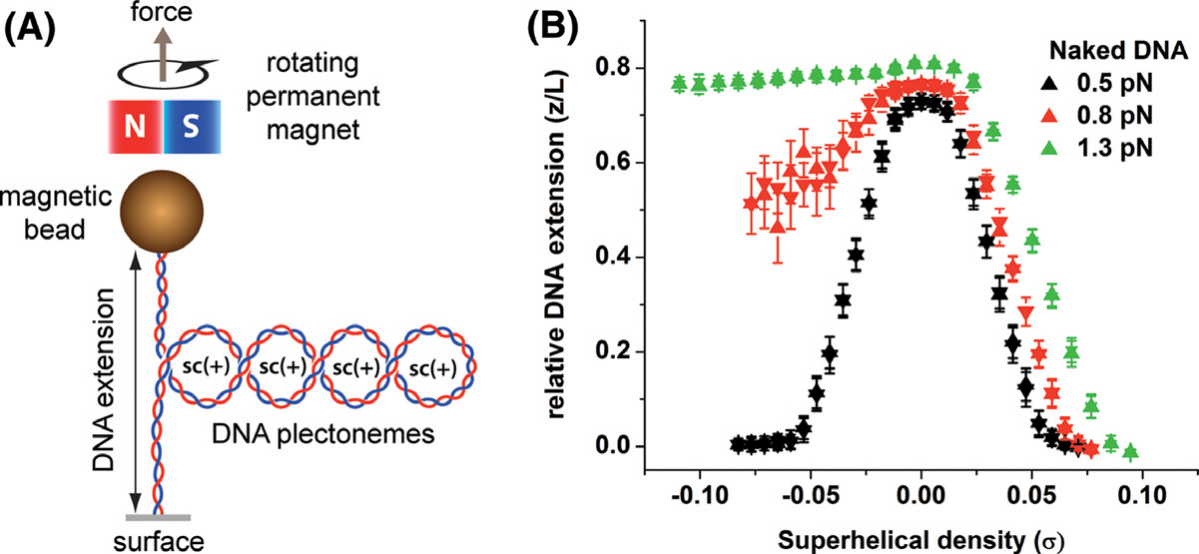
Single-DNA stretching and winding/unwinding using magnetic tweezers. (A) A micron-sized paramagnetic bead is tethered to a functionalized glass surface via a single DNA molecule which is multiply-anchored to form a torsionally constrained DNA tether. Translating or rotating the magnet allowed us to regulate the imposed DNA tension and linking number change. (B) TECs of a 7474 bp DNA (derived from pRL574, see Materials and Methods) used in this study held at various DNA tensions. Up-triangles represent increasing DNA winding (+*σ*)/unwinding (−*σ*), down-triangles represent reverse DNA winding/unwinding. All data points are mean values of DNA extension that are collected within a 10 s window and corresponding error bars are the standard deviation values of the DNA extension averaging.

Winding or unwinding of a torsionally constrained DNA tether by magnetic tweezers (Figure 1A) results in DNA linking number, *Lk to change.* Hence, DNA winding or unwinding results in a positive or negative change in DNA *Lk*, respectively (i.e. 1 turn of DNA winding/unwinding result in +/− Δ*Lk*). According to the White's theorem (Δ*Lk* = *Tw* +*Wr*), a change in *Lk* causes a corresponding change in DNA twists, *Tw* and writhes, *Wr*. Depending on the tension imposed along DNA backbone, the accumulated torsion stress can be relaxed through DNA chiral bending, resulting in DNA conformational changes. At sufficiently low DNA tension, DNA Δ*Lk* is mainly stored in *Wr*, causing DNA supercoiling. This leads to formation of either (+) plectonemes (left-handed) during winding or (−) plectonemes (right-handed) during unwinding (31). The onset of DNA plectoneme formation is indicated by the DNA buckling transition: beyond a threshold value of *σ* = *σ*_b_, additional DNA turns are rapidly converted to *Wr* to form DNA plectonemes. In this region, DNA extension is linearly dependent on the number of Δ*Lk* introduced (Figure 1B) and shows symmetric behavior on either side of turns (32–34). At higher DNA tension (such as 0.8 pN), the symmetry between DNA winding and unwinding is broken. This is because high DNA tension leads to a higher energy cost for chiral bending, most of the Δ*Lk* remains as *Tw* and thus causes DNA to melt during DNA unwinding. However, during DNA winding, formation of (+) plectonemes still occurs up to ∼5 pN (33). These previous observations were reproduced on a torsionally constrained DNA in our experimental setup as shown in Figure 1B.

### H-NS DNA-stiffening mode suppresses DNA plectoneme formation during DNA winding

At low magnesium conditions (≤2 mM), H-NS forms a rigid filament along DNA, causing DNA-stiffening to predominate (20,23,26). In previous single-DNA stretching experiments using torsionally unconstrained DNA, the H-NS DNA-stiffening mode resulted in an increase in DNA bending rigidity (20,23,26). Here, we examined the effects of H-NS DNA-stiffening mode on a torsionally constrained DNA tether by using magnetic tweezers to measure the DNA FEC. To increase the probability of finding a nick-free DNA tether during our magnetic tweezers experiments, we used a relatively shorter DNA tether (∼7.5 kb, derived from plasmid pRL574) as compared to the λ DNA tether (∼48 kb) that was used in previous H-NS single-molecule force-manipulation studies (20,23,26,35).

**Figure 2.**
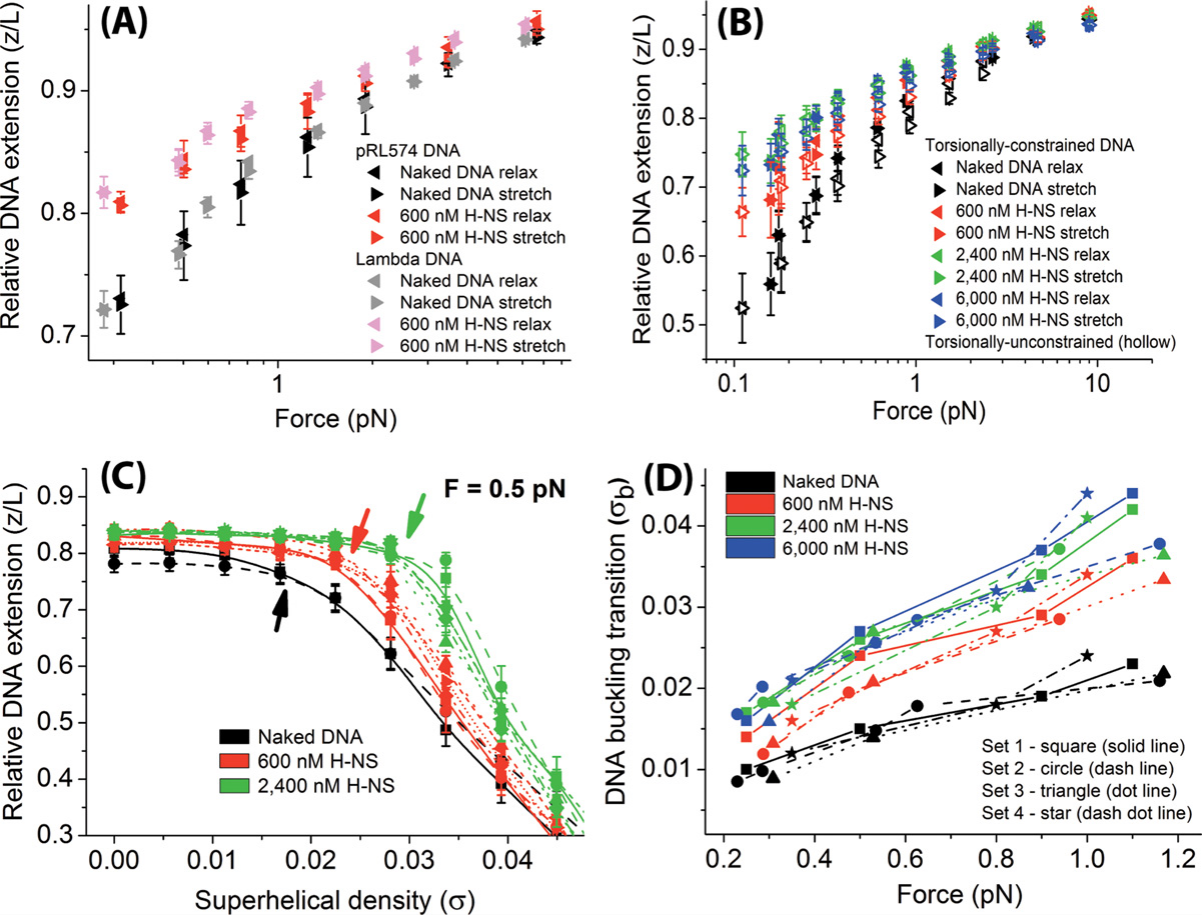
H-NS DNA-stiffening mode suppresses the DNA buckling transition. (A) DNA FECs of pRL574 and λ DNA tether incubated in 600 nM H-NS in stiffening buffer. DNA was held for 10 s (30 s for λ DNA) during which the average extension was measured. The left-triangles represent force-decrease scan data while the right triangles represent the subsequent force-increase scan data. (B) FECs of torsionally constrained (solid symbols) or unconstrained (hollow symbols) pRL574 DNA tether in presence of 600, 2400 and 6000 nM H-NS in stiffening buffer. (C) DNA TEC in the presence of 600 and 2400 nM H-NS in DNA-stiffening mode suppressed DNA buckling transition during DNA winding. DNA turns or Δ*Lk* are represented as superhelical density (*σ*). The data are multiple overlay of independent data sets (*N* = 2 for naked DNA data & *N* = 6 for H-NS data). Different symbols and connecting lines are used for different data sets: square (solid) – set 1; circle (dash) – set 2; up-triangle (dot) – set 3; diamond (dash dot dot) – set 4; left-triangle (short dash) – set 5 and right-triangle (short dot) – set 6. The data points and error bars are mean values of DNA extension and its standard deviation, respectively. The individual threshold superhelical density for DNA buckling transitions (*σ*_b_) are indicated by the arrows of the respective colors. (D) The increase in DNA buckling transition (*σ*_b_) was plotted against a DNA tension range of 0.2–1.2 pN in 600 (red), 2400 (green) and 6000 nM (blue) H-NS in stiffening buffer. The plot represents four independent data sets indicated by indicated solid symbols at each H-NS concentration.

We found that the FECs of torsionally unconstrained pRL574 and λ DNA tethers, measured in the stiffening buffer (10 mM Tris, 50 mM KCl, pH 7.5) in the presence of 600 nM H-NS, are similar to each other (Figure 2A). This indicates that in our experimental conditions, the H-NS DNA-stiffening mode is not significantly affected by DNA sequences or length. Next, we measured the FECs of both torsionally unconstrained (Figure 2B, hollow triangles) and torsionally constrained (Figure 2B, filled triangles) pRL574 DNA tether at *σ* = 0 in the same experimental condition with varying H-NS concentrations. Both FECs measurements from the two constructs nearly overlap each other. This suggests that H-NS polymerization along DNA does not produce significant DNA linking number change. In 50 mM KCl salt condition, 600 nM of H-NS was near saturation of H-NS DNA-stiffening effect while ≥2400 nM H-NS reached saturation.

To investigate how H-NS DNA-stiffening mode affects DNA plectonemes formation upon DNA winding (Δ*Lk*, also *σ* > 0), we wound the DNA tether at a tension of 0.5 pN at 600 and 2400 nM H-NS (red and green data sets, respectively), starting from a torsionally relaxed DNA state (Δ*Lk*, also *σ* = 0) (Figure 2C). Multiple independent data sets (*N* = 2 for naked DNA, *N* = 6 for 600 nM and 2400 nM H-NS) were overlaid in Figure 2C to demonstrate experimental repeatability. During DNA winding, (+) plectonemes were formed but with the DNA buckling transition happening at a higher *σ* value as compared to naked DNA. The measured DNA buckling transition (for method, see Supplementary Figure S1) for naked DNA at 0.5 pN was estimated to occur at *σ* ≈ 0.016 (Figure 2C, black arrow). In the presence of 600 nM H-NS, the DNA buckling transition upon DNA winding was increased to *σ* ≈ 0.023 (Figure 2C, red triangles and arrow). Further increasing H-NS concentration to 2.4 μM H-NS—where H-NS DNA-stiffening reaches saturation—caused a further increase in DNA buckling transition at *σ* ≈ 0.030 (Figure 2C, green triangles and arrow). This confirms the suppression of DNA buckling transition is caused by H-NS DNA-stiffening.

To get a quantitative representation of the suppression of DNA buckling transition by H-NS DNA-stiffening mode during DNA winding, we measured the threshold superhelical density (*σ*_b_) where DNA buckling transition occurred, during DNA winding in various H-NS concentrations and across a range of DNA tension (Figure 2D). It is clear that H-NS in DNA-stiffening mode consistently caused an increase in *σ*_b_ over a range of DNA tension and the effect was saturated at 2.4 μM H-NS. DNA buckling studies for DNA unwinding was not performed due to complications from DNA torsion-melting at higher DNA tension.

### H-NS DNA-stiffening mode impedes DNA supercoiling relaxation from plectonemic conformation

**Figure 3.**
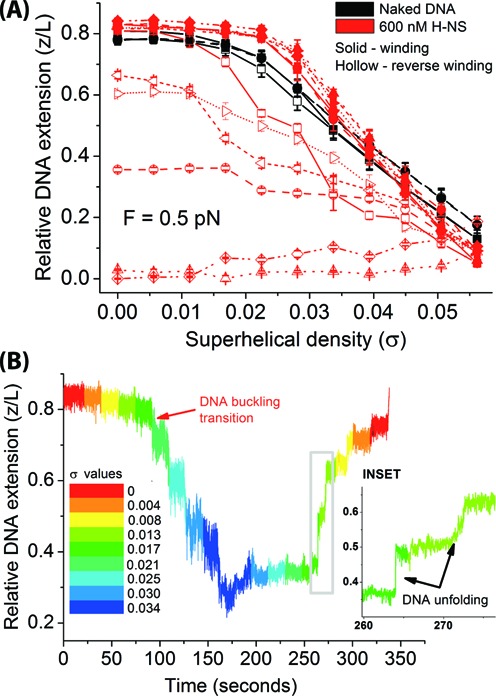
The H-NS DNA-stiffening mode restricts DNA conformational changes upon DNA linking number changes. (A) DNA extension hysteresis was observed between DNA winding (red solid) and reverse DNA winding (red hollow) in the presence of 600 nM H-NS bound to DNA in stiffening buffer. Six independent data sets are overlaid in the same plot, which are indicated by different symbols and connecting lines: square (solid) – set 1; circle (dash) – set 2; up-triangle (dot) – set 3; diamond (dash dot dot) – set 4; left-triangle (short dash) – set 5 and right-triangle (short dot) – set 6. Data point and error bar represents mean DNA extension and its standard deviation, respectively. (B) Time-course showed DNA extension cannot be recovered immediately upon reverse winding of (+) supercoiled DNA in the presence of H-NS, resulting in a large hysteresis. Inset: unfolding of a transiently trapped (+) plectoneme conformation of DNA by H-NS DNA-stiffening mode at constant *σ*.

Winding of DNA bound with H-NS in the stiffening mode at 0.5 pN delayed the buckling transition as demonstrated in Figure 2C and D and also in Figure 3A. Plectoneme formation eventually followed after buckling, but when we attempted to remove the introduced (+) Δ*Lk* from the prior DNA winding process (this process defined as reverse winding hereafter), we observed a large hysteresis in DNA extension. The hysteresis is defined by the extension difference between the DNA winding (Figure 3A, solid red symbols) and the reverse winding curves (Figure 3A, hollow red symbols), where the reverse winding curve had a lower DNA extension. To demonstrate the repeatability of the experiment and the stochastic nature of the observed hysteresis, we plotted the overlay of six independent data sets. Similar hysteresis was also observed when we increased H-NS concentration to 6 μM where H-NS DNA-stiffening effect is saturated (Supplementary Figure S2). Such hysteresis indicates that the relaxation of DNA supercoiling from plectonemes was impeded by H-NS bound on DNA in the stiffening mode.

Closer examination of the kinetics revealed that during DNA winding, at each constant *σ* value, no progressive reduction in DNA extension was observed (Figure 3B). This was confirmed by independent experiments on a longer time scale when we held DNA at high *σ* values for up to 20 min (Supplementary Figure S3). These results suggest that the cause of the observed DNA extension hysteresis in Figure 3A is not the result of H-NS-mediated DNA-bridging as observed in presence of high magnesium (23), which causes progressive DNA folding and would expected to lead to progressive DNA extension decrease at a constant *σ*. Further, the time trace during reverse winding revealed several DNA unfolding signals that were mixed with gradual and step-wise DNA extension increases when DNA was held at constant *σ* values (Figure 3B, inset).

Taking together this result with the previous section result, it suggests that H-NS in the stiffening-binding mode tends to maintain the current topological/conformational state of DNA; H-NS-bound linear DNA at low superhelical density (*σ*) disfavors (+) plectoneme formation during DNA winding. However, after (+) plectonemes are formed, H-NS stabilizes them against torsion relaxation (Δ*Lk* relaxation) during DNA reverse winding (see Discussion section for the possible implications and mechanisms).

### H-NS DNA-stiffening mode causes DNA melting-like behavior during DNA unwinding

**Figure 4.**
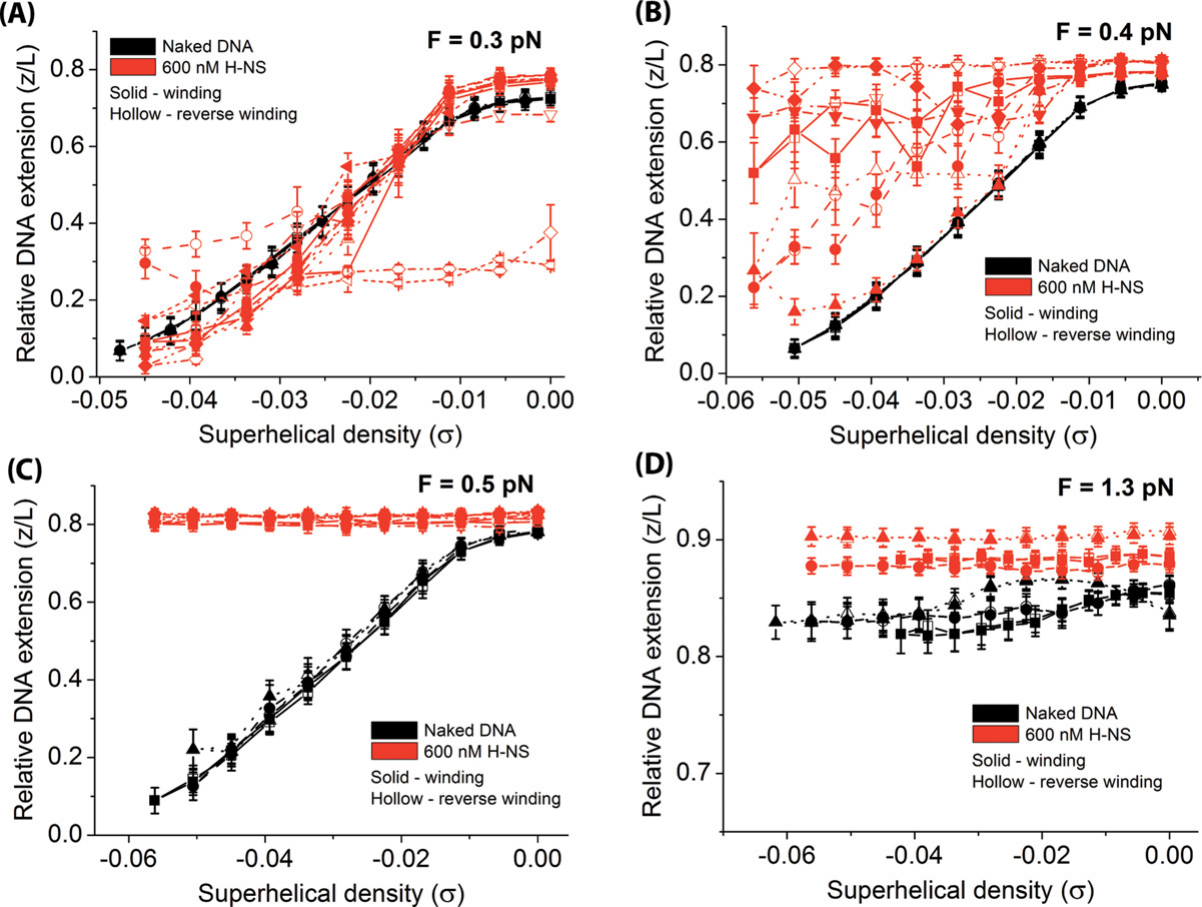
The H-NS DNA-stiffening mode promotes DNA melting-like behavior during DNA unwinding. The plots are overlaid with six independent data sets: square (solid) – set 1; circle (dash) – set 2; up-triangle (dot) – set 3; down-triangle (dash dot) – set 4; diamond (dash dot dot) – set 5 and left-triangle (short dash) – set 6. (A) At 0.3 pN, (−) DNA plectoneme formed with hysteresis during DNA reverse unwinding. (B) At 0.4 pN, in the presence of H-NS DNA-stiffening, H-NS-bound DNA exhibited partially DNA melted-like behavior while the naked DNA still formed (−) plectonemes. (C) At 0.5 pN, H-NS-bound DNA showed complete DNA melted-like behavior while DNA still formed (−) plectonemes. (D) At 1.3 pN, both naked DNA and H-NS-bound DNA showed complete melting during DNA unwinding, although the DNA extension in the presence of H-NS is slightly higher than the naked DNA extension.

Next, we performed turns-extension measurements of the DNA bound with H-NS in the DNA-stiffening mode during DNA unwinding (Δ*Lk* < 0) followed by reverse unwinding (removing prior introduced (−) Δ*Lk*), done at a range of DNA tensions (Figure 4). Again, we plotted the overlay of six independent data sets to demonstrate repeatability of experiments. The individual data set is represented by the symbols indicated in Figure 4 legend. At a DNA tension of 0.3 pN (Figure 4A), (−) plectonemes were formed (linear decrease in DNA extension with DNA unwinding) for naked DNA (black symbols) and H-NS-bound DNA (red symbols). However, occasionally, we saw that H-NS-bound DNA exhibited DNA extension hysteresis between the DNA unwinding (solid symbols) and DNA reverse unwinding curves (hollow symbols), similar to what was observed in Figure 3A.

When the DNA tension was slightly increased to 0.4 pN (Figure 4B), the H-NS-bound curve exhibited a much higher DNA extension than naked DNA and also with larger hysteresis. However, naked DNA still formed (−) plectonemes. The H-NS-bound DNA data are reminiscent to that of a partially melted DNA curve albeit measured at higher DNA tensions (i.e. see Figure 1B, 0.8 pN curve). This suggests H-NS DNA stiffening effect may promote DNA melting during DNA unwinding, similar to the effect of increasing DNA tension. Indeed, when we further increased the DNA tension to 0.5 pN (Figure 4C), H-NS-bound DNA has the same result as a completely melted DNA during unwinding—whereby the DNA extension does not significantly change as the DNA was increasingly unwound (i.e. see Figure 1B, 1.3 pN curve and Figure 4D, black symbols). For naked DNA, (−) plectonemes was still formed.

Further increasing the DNA tension to 1.3 pN resulted in similar results for both naked DNA and H-NS-bound DNA (Figure 4D), whereby DNA extension was not significantly changed during DNA unwinding. The strong resemblance of H-NS-bound DNA data at a lower tension to that of a melted naked DNA from unwinding at higher DNA tension supports that H-NS DNA-stiffening mode likely promotes DNA melting during DNA unwinding, at DNA tensions that would otherwise result in (−) plectonemes formation in the absence of H-NS. In fact, this can be easily be explained by the higher energy cost in bending DNA caused by H-NS DNA-stiffening mode (see Discussion). We saw similar DNA melting-like behavior when the experiments were repeated with 6 μM H-NS (Supplementary Figure S4).

We also noted that whenever there was partial DNA melting-like behavior during DNA unwinding in the presence of H-NS, a larger hysteresis in DNA extension between unwinding and reverse unwinding was observed than that observed on partial melting of a naked DNA induced by higher tension (e.g. compare Figure 4B, red symbols with Figure 1B, red symbols). The significance of these observations is unclear, but they may be related to H-NS single-stranded DNA (ssDNA) binding during melting as H-NS is known to bind to ssDNA although with a lower affinity than to dsDNA (36).

### H-NS DNA-bridging mode promotes DNA plectoneme formation and blocks twist diffusion

At higher magnesium conditions (i.e. >5 mM MgCl_2_), DNA-bridging is the predominant form of DNA-binding (21,23). At 10 mM MgCl_2_, the H-NS DNA-stiffening effect is effectively abolished and H-NS DNA-bridging dominates (23). In this section, we investigate the impact of the H-NS DNA-bridging binding mode on DNA supercoiling formation at 10 mM MgCl_2_.

**Figure 5.**
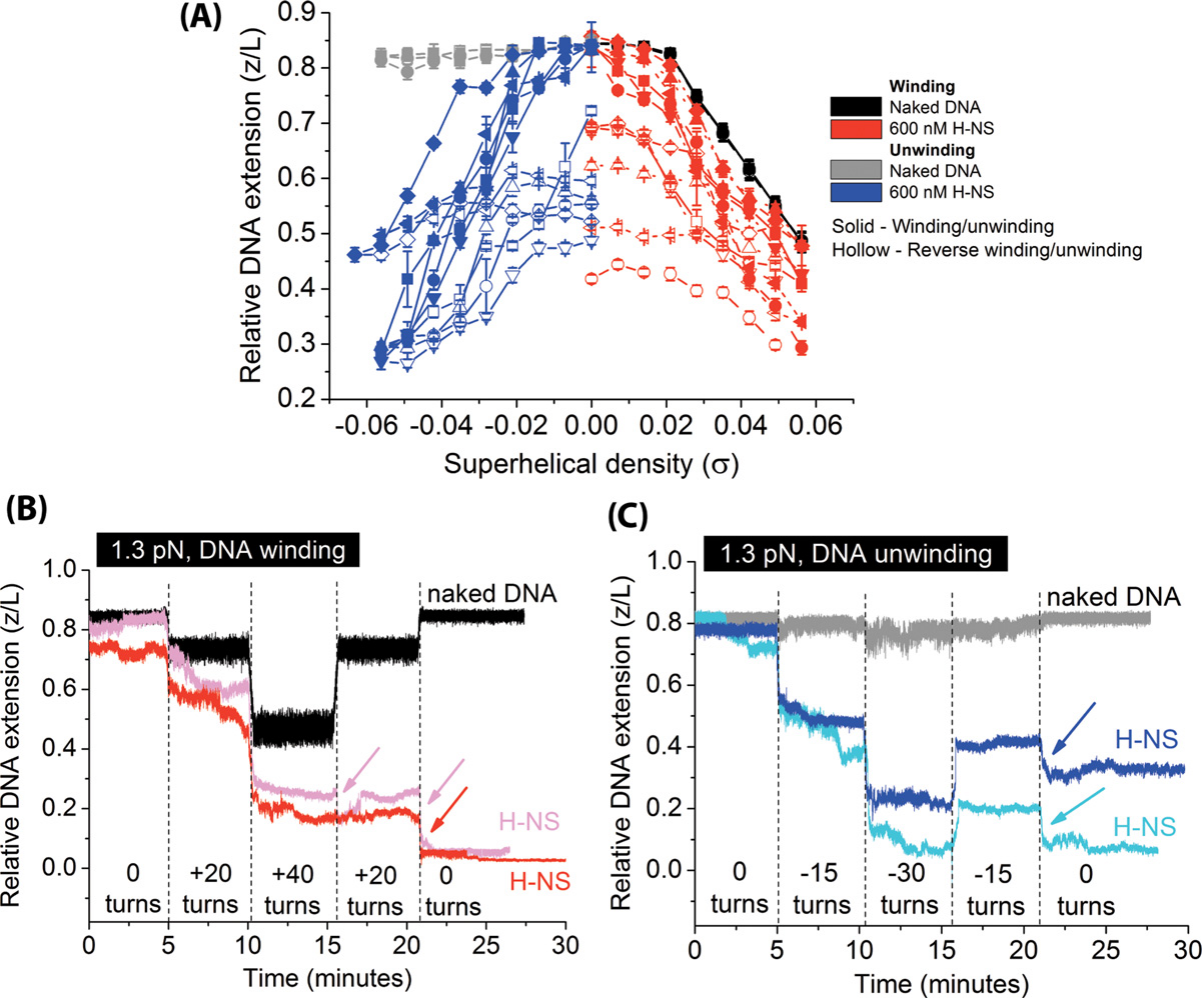
The H-NS DNA-bridging mode promotes DNA plectoneme formation and blocks twist diffusion. (A) DNA turns-extension curves done at 1.3 pN in the presence of 600 nM H-NS in DNA-bridging buffer. Six independent data sets are overlaid in the same plot: square (solid) – set 1; circle (dash) – set 2; up-triangle (dot) – set 3; down-triangle (dash dot) – set 4; diamond (dash dot dot) – set 5 and left-triangle (short dash) – set 6. Black and gray triangles represent data obtained from winding/reverse winding (solid) and unwinding/reverse unwinding (hollow) of naked DNA, respectively. Red and blue triangles represent data obtained from winding/reverse winding (solid) and unwinding/reverse unwinding (hollow) of DNA in the presence of 600 nM H-NS, respectively. (B) Time-courses of extensions of DNA held at 1.3 pN in the absence of H-NS (black) and in the presence of 600 nM H-NS (two independent experiments indicated by pink and red colors) in DNA-bridging buffer during controlled DNA winding and reverse winding procedure. In the presence of H-NS, DNA extension was not fully recovered upon reverse winding and instead resulted in further reduction of DNA extension (see arrows). (C) A similar experiment to that in panel B was performed by unwinding followed by reverse unwinding of DNA showed similar behavior.

In this DNA-bridging condition, we found the effect of H-NS on DNA supercoiling is in sharp contrast to that of the H-NS DNA-stiffening mode. The DNA turns-extension curve in the presence of 600 nM H-NS in bridging buffer (10 mM Tris, 50 mM KCl, 10 mM MgCl_2_, pH 7.5) at DNA tension of 1.3 pN showed (+) plectoneme formation began immediately once the H-NS bound DNA was wound (Figure 5A, red solid symbols). This was in contrast to naked DNA where a certain amount of DNA winding is needed for buckling to occur to form (+) plectonemes (Figure 5A, black solid symbols). This is also in contrast to the effect of H-NS DNA stiffening mode which suppresses plectoneme formation during DNA winding. This result suggests that H-NS in its DNA-bridging mode promotes (+) plectoneme formation upon DNA winding.

After (+) plectonemes were formed, reverse winding could not recover the extension (Figure 5A, red hollow symbols). This hysteresis was caused by a different mechanism from the hysteresis that was observed in the H-NS stiffening mode as shown in Figure 3A. The hysteresis observed in the H-NS stiffening mode was not caused by any progressive DNA folding mechanism while in the H-NS DNA bridging mode, the extension drop during DNA winding was caused by progressive folding even when the DNA was held a constant positive *σ* and constant tension (Supplementary Figure S5). Such progressive folding is consistent with the large DNA extension hysteresis observed between DNA winding and reverse unwinding curves in Figure 5A, and indicates that H-NS DNA-bridging mode also traps DNA in plectonemes. As the DNA extension could not always be readily recovered after reverse winding to *σ* = 0 at DNA tension of 1.3 pN, we had to resort to using a higher DNA tension of >10 pN to unfold the H-NS-folded DNA to recover the DNA tether to its original extension before performing the next experiment.

Next, we investigated the effect of H-NS DNA-bridging mode to DNA unwinding and reverse unwinding in the presence of 600 nM H-NS at 1.3 pN. (−) DNA plectonemes were formed during unwinding (Figure 5A, blue solid symbols) and it appeared symmetric to the winding curve (compare to Figure 5A, red solid symbols). This result is drastically different from both naked DNA and H-NS-bond DNA in H-NS DNA stiffening mode where DNA exhibited DNA melting-like behavior during unwinding. It indicates that H-NS bridging mode has an effect of preventing DNA melting through promoting (−) plectoneme formation via DNA-bridging during DNA unwinding. As expected from DNA bridging by H-NS, a large hysteresis was also observed during the reverse unwinding process (Figure 5A, blue hollow symbols).

The results in Figure 5A are easily explained by H-NS-mediated DNA bridging properties. The H-NS DNA bridging mode naturally promotes DNA plectoneme formation through DNA-H-NS-DNA bridging, which reduces DNA bending energy cost during plectoneme formation. Overall, H-NS DNA-bridging assisted plectoneme formation leads to a reduction in DNA torsion stress during DNA winding or unwinding. This leads to the suppression of DNA melting during DNA unwinding in conditions where DNA melting would have occurred in the absence of H-NS DNA-bridging effect (see Discussion).

H-NS is proposed to play a role in forming topologically distinct domains (37), likely through blocking twist diffusion across domains. To test whether H-NS blocks twist diffusion, we first introduced (+/−) Δ*Lk* to the DNA in the presence of H-NS in bridging buffer, allowing H-NS DNA-bridging to stabilize the resulting DNA plectonemes. The introduced DNA (+/−) Δ*Lk* were then removed through either reverse DNA winding or reverse unwinding. If H-NS DNA-bridging can block twist diffusion, the H-NS stabilized DNA plectonemes would not be affected but rather, new DNA plectonemes of opposite-chirality would form and this can be detected by a sharp drop in DNA extension during DNA reverse winding/unwinding process.

Indeed, this prediction was observed in our experiments. Figure 5B shows the DNA extension time-trace during DNA winding as +20 DNA turns (Δ*Lk* = +20) were introduced at every interval of 5 min up to +40 turns before reversing the DNA winding process. At each winding step, a sharp extension drop was observed and then followed by a slower gradual extension reduction. This is consistent with spontaneous DNA plectoneme formation upon winding and then followed by further stabilization from H-NS DNA bridging. We observed that the DNA extension could not be recovered when DNA Δ*Lk* was reduced from +40 turns to +20 turns (reverse winding), suggesting that the prior-formed (+) plectonemes cannot be relaxed by reducing DNA Δ*Lk*. In addition, the DNA extension decreased sharply with a large step-size when the DNA Δ*Lk* was further reduced from +20 turns to 0 turns (Figure 5B, see red or pink arrow). Similar observations were seen in DNA unwinding experiments when DNA Δ*Lk* was reduced from −15 turns to 0 turns (Figure 5C, cyan or blue arrow, DNA reverse unwinding). These results were also observed at higher H-NS concentration when similar experiments were repeated (Supplementary Figure S6).

## DISCUSSION

**Figure 6.**
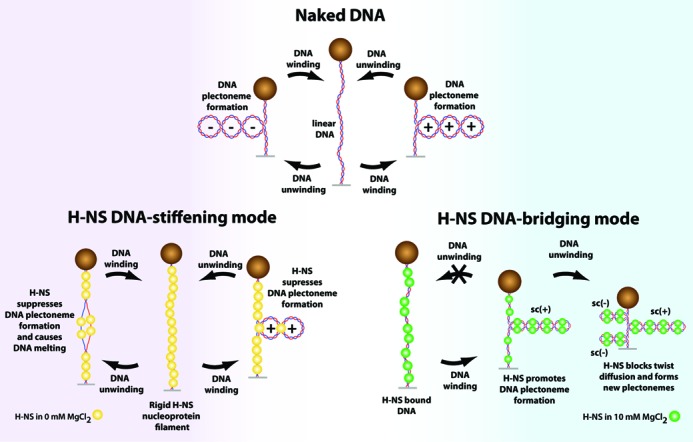
Summary of the proposed H-NS DNA-binding modes effects on DNA supercoiling. DNA unwinding or winding results in naked DNA plectoneme formation at the indicated DNA tension. With H-NS bound to DNA in the stiffening mode, the increase in DNA bending rigidity causes DNA melting instead of DNA (−) plectoneme formation during DNA unwinding. During DNA winding, DNA (+) plectoneme formation is suppressed. For H-NS bound to DNA in the bridging mode, H-NS promotes DNA plectoneme formation through stabilizing DNA plectoneme formation by DNA bridging. H-NS DNA-bridging also blocks twist diffusion demonstrated by the formation of new DNA plectonemes of opposite supercoiling chirality when the DNA linking number was reduced.

Most of the single-molecule studies on NAPs DNA-binding properties have been performed using torsionally unconstrained DNA (non-supercoilable DNA) (20,38–40). Apart from this work, only one other single-molecule study addressed how the NAP HU (a DNA bending protein) from *Bacillus stearothermophilus* can affect DNA supercoiling (41). Our work shows for the first time how the DNA supercoiling state is differentially regulated by H-NS based on its two distinct DNA-binding modes—DNA-stiffening favored at low magnesium concentrations and DNA-bridging favored at high magnesium concentrations (Figure 6). Two magnesium concentrations, 0 and 10 mM, were chosen to prevent co-existence of the two binding modes so that the effects of individual binding modes on DNA supercoiling can be studied.

The DNA-stiffening mode is a result of formation of an H-NS nucleoprotein filament that restricts DNA bending. In single-DNA stretching measurements, it leads to an increase in the apparent DNA-bending persistence length (20,23,26). Therefore, it results in a higher energy cost in forming the curved DNA plectonemes. This helps explain why the H-NS DNA-stiffening mode can suppress DNA plectoneme formation during DNA winding and why it promotes DNA melting during DNA unwinding at DNA tensions where (−) plectonemes would otherwise form in the absence of H-NS. One peculiar finding is that once DNA plectonemes are formed (at higher DNA Δ*Lk*), the H-NS DNA-stiffening mode is able to passively trap DNA in its supercoiled conformation, which is not directly expected from its DNA stiffening effect. Collectively, these observations indicate that H-NS rigid nucleoprotein filament can impede DNA conformational changes during DNA linking number changes.

Although the cause of DNA plectoneme trapping by H-NS DNA-stiffening mode is unclear, its passive nature is different from that of the magnesium-induced H-NS DNA-bridging, which causes progressive folding (23,42). One speculative model is that H-NS binding in the stiffening mode can adapt into a rigid helical filament, which helical axis follows the DNA double-helix backbone inside the plectonemes. It would then resists to supercoiling relaxation during reverse winding or reverse unwinding, a process that requires a much extended/linear DNA conformation.

In the presence of 10 mM MgCl_2_, whereby the H-NS DNA-bridging mode predominates (23), H-NS promoted DNA plectoneme formation. This is expected, as the close-proximity DNA strands at the loop apex and base would allow DNA-H-NS-DNA bridges—mediated by either H-NS dimer (35) or by an helical H-NS scaffold proposed recently (22)—to nucleate and progressively zip up the DNA loop and leads to stabilization of DNA plectonemes conformation. It is also possible that H-NS DNA-bridging would further ‘zip up’ the DNA segments branching from plectoneme loop base. Energetically, H-NS DNA-bridging reduces the energy cost in DNA plectoneme formation and maintenance during DNA winding or unwinding. This also explains why DNA melting was suppressed during DNA unwinding at high DNA tension, as plectoneme formation reduces DNA torsion stress.

The large, sharp decreases in DNA extension that were observed during the processes of reverse winding or reverse unwinding of pre-formed (+/-) DNA plectonemes in the presence of H-NS DNA-bridging (Figure 5B and C) are unlikely due to spontaneous folding of DNA by H-NS as we only observed gradual DNA extension decreases when the DNA Δ*Lk* was kept constant (Figure 5B and C, note DNA extension time-trace at constant *σ*) and the DNA tension used in Figure 5B and C is too high for H-NS to bridge large DNA-loops—which would caused a sharp decrease in DNA. Rather, such sharp and large extension decreases can be naturally explained by the formation of new DNA plectonemes of opposite chirality since the direction of DNA Δ*Lk* change was reversed. This implies that when H-NS is bound to DNA in its DNA-bridging mode, it likely forms a barrier to twist diffusion and leads to the formation of topologically isolated supercoiled DNA domains.

This work thus represents the first direct mechanical evidence supporting that H-NS behaves as a domainin, as proposed in a previous *in vivo* study (37). Previously, two models of constraining DNA supercoiling topology by DNA-binding proteins were proposed—DNA-looping (DNA-bridging in our context) and DNA-wrapping specific proteins are capable of forming supercoiling insulating nucleoprotein complexes (43,44). The ‘DNA-looping protein model’ was supported by biochemical assays showing that highly specific DNA-looping proteins, such as the *lac* repressor protein that can induce DNA linking number changes (45), and also block supercoiling diffusion through forming DNA-bridges at the base of the supercoiled DNA domain (44). Hence, our results provide mechanical evidences to further support the proposed model, by showing that non-specific DNA binding NAPs have the ability to act as a DNA topological insulator through non-specific DNA-bridging actions. Together, these results highlight the importance of the DNA architectural properties of DNA-binding proteins to their roles in organizing supercoiled domains in bacterial chromosomes.

H-NS is a global gene regulator, mostly repressing genes (14,16–18). Previously, the possible gene-silencing mechanisms of H-NS were mainly based on either blocking access of RNA polymerase (RNAP) to promoter sites or impeding the translocation of RNAP during elongation phase (24–26,46). The findings from this work provide new possible mechanisms based on H-NS's effects on DNA supercoiling. RNAP generates (+) supercoiling downstream and the amount of supercoiling accumulated can regulate RNAP activity (47,48). Indeed, an *in vitro* study on torque regulation of RNAP translocation activity showed DNA torque can stall RNAP (48). As such, one possible novel gene silencing mechanism by H-NS is that the formation of H-NS nucleoprotein filament downstream of promoter sites can increase the level of DNA torque by increasing the energy cost of DNA buckling transition to stall RNAP during elongation. Alternatively, H-NS DNA-bridging mode constraining of DNA in plectonemes and preventing of twist diffusion suggests it can cause accumulation of DNA torsion stress in front of RNAP, which may also lead to RNAP activity inhibition.

This work also provides a demonstration that DNA-stiffening proteins should suppress DNA plectonemes formation and promote DNA melting during unwinding due to an increase in DNA-bending rigidity. DNA-bridging proteins have opposite effects by promoting DNA plectonemes formation and suppressing melting during DNA unwinding. Given that DNA-stiffening, DNA-bridging/looping and DNA-bending/wrapping behaviors have been reported for numerous bacterial NAPs (49–54), our findings obtained from the two distinct DNA-binding modes of H-NS and together with the previous *B. stearothermophilus* HU study (41), suggest that the DNA architectural proteins may employ their distinct DNA-binding modes to passively regulate the supercoiling state of the bacterial nucleoid which may directly or indirectly affect chromosomal DNA organization dynamics and gene regulation. Henceforth, understanding the interplay between NAPs and their gene regulatory and chromosomal packaging roles in the context of DNA supercoiling is important, which provides a more physiologically relevant platform to understand how bacterial NAPs performs their biological functions *in vivo*.

## Authors Contributions

C.J.L. performed the experiments. C.J.L., L.J.K. and J.Y. conceived the research. C.J.L., L.J.K. and J.Y. designed the experiments. C.J.L. and J.Y. analyzed and interpreted the data. C.J.L., L.J.K. and J.Y. wrote the paper.

## SUPPLEMENTARY DATA

Supplementary Data are available at NAR Online.

SUPPLEMENTARY DATA

## References

[1] Fuller F.B. (1971). The writhing number of a space curve. Proc. Natl. Acad. Sci. U.S.A..

[2] White J.H. (1969). Self-linking and the Gauss integral in higher dimensions. Am. J. Math..

[3] Miller W.G., Simons R.W. (1993). Chromosomal supercoiling in Escherichia coli. Mol. Microbiol..

[4] Pang Z.H., Chen R., Manna D., Higgins N.P. (2005). A gyrase mutant with low activity disrupts supercoiling at the replication terminus. J. Bacteriol..

[5] Baker T.A., Sekimizu K., Funnell B.E., Kornberg A. (1986). Extensive unwinding of the plasmid template during staged enzymatic initiation of DNA-replication from the origin of the Escherichia coli chromosome. Cell.

[6] Pruss G.J., Drlica K. (1989). DNA supercoiling and prokaryotic transcription. Cell.

[7] Higgins C.F., Dorman C.J., Stirling D.A., Waddell L., Booth I.R., May G., Bremer E. (1988). A physiological role for DNA supercoiling in the osmotic regulation of gene expression in S. typhimurium and E. coli. Cell.

[8] Wu H.Y., Shyy S.H., Wang J.C., Liu L.F. (1988). Transcription generates positively and negatively supercoiled domains in the template. Cell.

[9] Stuger R., Woldringh C.L., van der Weijden C.C., Vischer N.O.E., Bakker B.M., van Spanning R.J.M., Snoep J.L., Westerhoff H.V. (2002). DNA supercoiling by gyrase is linked to nucleoid compaction. Mol. Biol. Rep..

[10] Sinden R.R., Pettijohn D.E. (1981). Chromosomes in living Escherichia coli cells are segregated into domains of supercoiling. Proc. Natl. Acad. Sci.-Biol..

[11] Wang X.D., Llopis P.M., Rudner D.Z. (2013). Organization and segregation of bacterial chromosomes. Nat. Rev. Genet..

[12] Dillon S.C., Dorman C.J. (2010). Bacterial nucleoid-associated proteins, nucleoid structure and gene expression. Nat. Rev. Microbiol..

[13] Browning D.F., Grainger D.C., Busby S.J. (2010). Effects of nucleoid-associated proteins on bacterial chromosome structure and gene expression. Curr. Opin. Microbiol..

[14] Hulton C.S., Seirafi A., Hinton J.C., Sidebotham J.M., Waddell L., Pavitt G.D., Owen-Hughes T., Spassky A., Buc H., Higgins C.F. (1990). Histone-like protein H1 (H-NS), DNA supercoiling, and gene expression in bacteria. Cell.

[15] Tupper A.E., Owen-Hughes T.A., Ussery D.W., Santos D.S., Ferguson D.J., Sidebotham J.M., Hinton J.C., Higgins C.F. (1994). The chromatin-associated protein H-NS alters DNA topology in vitro. EMBO J..

[16] McGovern V., Higgins N.P., Chiz R.S., Jaworski A. (1994). H-NS over-expression induces an artificial stationary phase by silencing global transcription. Biochimie.

[17] Ueguchi C., Mizuno T. (1993). The Escherichia coli nucleoid protein H-NS functions directly as a transcriptional repressor. EMBO J..

[18] Atlung T., Ingmer H. (1997). H-NS: a modulator of environmentally regulated gene expression. Mol. Microbiol..

[19] Lucchini S., Rowley G., Goldberg M.D., Hurd D., Harrison M., Hinton J.C. (2006). H-NS mediates the silencing of laterally acquired genes in bacteria. PLoS Pathog..

[20] Amit R., Oppenheim A.B., Stavans J. (2003). Increased bending rigidity of single DNA molecules by H-NS, a temperature and osmolarity sensor. Biophys. J..

[21] Dame R.T., Wyman C., Goosen N. (2000). H-NS mediated compaction of DNA visualised by atomic force microscopy. Nucleic Acids Res..

[22] Arold S.T., Leonard P.G., Parkinson G.N., Ladbury J.E. (2010). H-NS forms a superhelical protein scaffold for DNA condensation. Proc. Natl. Acad. Sci. U.S.A..

[23] Liu Y., Chen H., Kenney L.J., Yan J. (2010). A divalent switch drives H-NS/DNA-binding conformations between stiffening and bridging modes. Genes Dev..

[24] Dame R.T., Wyman C., Wurm R., Wagner R., Goosen N. (2002). Structural basis for H-NS-mediated trapping of RNA polymerase in the open initiation complex at the rrnB P1. J. Biol. Chem..

[25] Lang B., Blot N., Bouffartigues E., Buckle M., Geertz M., Gualerzi C.O., Mavathur R., Muskhelishvili G., Pon C.L., Rimsky S. (2007). High-affinity DNA binding sites for H-NS provide a molecular basis for selective silencing within proteobacterial genomes. Nucleic Acids Res..

[26] Lim C.J., Lee S.Y., Kenney L.J., Yan J. (2012). Nucleoprotein filament formation is the structural basis for bacterial protein H-NS gene silencing. Sci. Rep..

[27] Walthers D., Li Y., Liu Y.J., Anand G., Yan J., Kenney L.J. (2011). Salmonella enterica response regulator SsrB relieves H-NS silencing by displacing H-NS bound in polymerization mode and directly activates transcription. J. Biol. Chem..

[28] Maurer S., Fritz J., Muskhelishvili G. (2009). A systematic in vitro study of nucleoprotein complexes formed by bacterial nucleoid-associated proteins revealing novel types of DNA organization. J. Mol. Biol..

[29] Adelman K., La Porta A., Santangelo T.J., Lis J.T., Roberts J.W., Wang M.D. (2002). Single molecule analysis of RNA polymerase elongation reveals uniform kinetic behavior. Proc. Natl. Acad. Sci. U.S.A..

[30] Schlingman D.J., Mack A.H., Mochrie S.G.J., Regan L. (2011). A new method for the covalent attachment of DNA to a surface for single-molecule studies. Colloid Surface B.

[31] Strick T.R., Allemand J.F., Bensimon D., Bensimon A., Croquette V. (1996). The elasticity of a single supercoiled DNA molecule. Science.

[32] Neukirch S. (2004). Extracting DNA twist rigidity from experimental supercoiling data. Phys. Rev. Lett..

[33] Marko J.F. (2007). Torque and dynamics of linking number relaxation in stretched supercoiled DNA. Phys. Rev. E Stat. Nonlin. Soft. Matter Phys..

[34] Neukirch S., Marko J.F. (2011). Analytical description of extension, torque, and supercoiling radius of a stretched twisted DNA. Phys. Rev. Lett..

[35] Dame R.T., Noom M.C., Wuite G.J. (2006). Bacterial chromatin organization by H-NS protein unravelled using dual DNA manipulation. Nature.

[36] Zhu B., Lee S.J., Tan M., Wang E.D., Richardson C.C. (2012). Gene 5.5 protein of bacteriophage T7 in complex with Escherichia coli nucleoid protein H-NS and transfer RNA masks transfer RNA priming in T7 DNA replication. Proc. Natl. Acad. Sci. U.S.A..

[37] Hardy C.D., Cozzarelli N.R. (2005). A genetic selection for supercoiling mutants of Escherichia coli reveals proteins implicated in chromosome structure. Mol. Microbiol..

[38] Laurens N., Driessen R.P., Heller I., Vorselen D., Noom M.C., Hol F.J., White M.F., Dame R.T., Wuite G.J. (2012). Alba shapes the archaeal genome using a delicate balance of bridging and stiffening the DNA. Nat. Commun..

[39] Xiao B., Johnson R.C., Marko J.F. (2010). Modulation of HU-DNA interactions by salt concentration and applied force. Nucleic Acids Res..

[40] Lim C.J., Lee S.Y., Teramoto J., Ishihama A., Yan J. (2013). The nucleoid-associated protein Dan organizes chromosomal DNA through rigid nucleoprotein filament formation in E. coli during anoxia. Nucleic Acids Res..

[41] Schnurr B., Vorgias C., Stavans J. (2006). Compaction and supercoiling of single, long DNA molecules by HU protein. Biophys. Rev. Lett..

[42] Wiggins P.A., Dame R.T., Noom M.C., Wuite G.J.L. (2009). Protein-mediated molecular bridging: a key mechanism in biopolymer organization. Biophys. J..

[43] Fulcrand G., Zhi X., Leng F. (2013). Transcription-coupled DNA supercoiling in defined protein systems and in E. coli topA mutant strains. IUBMB Life.

[44] Leng F., Chen B., Dunlap D.D. (2011). Dividing a supercoiled DNA molecule into two independent topological domains. Proc. Natl. Acad. Sci. U.S.A..

[45] Chen B., Xiao Y., Liu C., Li C., Leng F. (2010). DNA linking number change induced by sequence-specific DNA-binding proteins. Nucleic Acids Res..

[46] Shin M., Song M., Rhee J.H., Hong Y., Kim Y.J., Seok Y.J., Ha K.S., Jung S.H., Choy H.E. (2005). DNA looping-mediated repression by histone-like protein H-NS: specific requirement of Esigma70 as a cofactor for looping. Genes Dev..

[47] Liu L.F., Wang J.C. (1987). Supercoiling of the DNA template during transcription. Proc. Natl. Acad. Sci. U.S.A..

[48] Ma J., Bai L., Wang M.D. (2013). Transcription under torsion. Science.

[49] van Noort J., Verbrugge S., Goosen N., Dekker C., Dame R.T. (2004). Dual architectural roles of HU: formation of flexible hinges and rigid filaments. Proc. Natl. Acad. Sci. U.S.A..

[50] Lim C.J., Whang Y.R., Kenney L.J., Yan J. (2012). Gene silencing H-NS paralogue StpA forms a rigid protein filament along DNA that blocks DNA accessibility. Nucleic Acids Res..

[51] Winardhi R.S., Fu W., Castang S., Li Y., Dove S.L., Yan J. (2012). Higher order oligomerization is required for H-NS family member MvaT to form gene-silencing nucleoprotein filament. Nucleic Acids Res..

[52] Chen J.M., Ren H., Shaw J.E., Wang Y.J., Li M., Leung A.S., Tran V., Berbenetz N.M., Kocincova D., Yip C.M. (2008). Lsr2 of Mycobacterium tuberculosis is a DNA-bridging protein. Nucleic Acids Res..

[53] Qu Y., Lim C.J., Whang Y.R., Liu J., Yan J. (2013). Mechanism of DNA organization by Mycobacterium tuberculosis protein Lsr2. Nucleic Acids Res..

[54] Ali B.M.J., Amit R., Braslavsky I., Oppenheim A.B., Gileadi O., Stavans J. (2001). Compaction of single DNA molecules induced by binding of integration host factor (IHF). Proc. Natl. Acad. Sci. U.S.A..

