# Supplementary material for: Single-molecule studies on the mechanical interplay between DNA supercoiling and H-NS DNA architectural properties
Source: Nucleic Acids Res. 2014 Jul 18;42(13):8369–78. doi: 10.1093/nar/gku566 (PMC4117784; doi:10.1093/nar/gku566)
Supplement: SUPPLEMENTARY DATA [file supp_42_13_8369__index.html]

Single-molecule studies on the mechanical interplay between DNA supercoiling and H-NS DNA architectural properties — Single-molecule studies on the mechanical interplay between DNA supercoiling and H-NS DNA architectural properties — SUPPLEMENTARY DATA 

# Single-molecule studies on the mechanical interplay between DNA supercoiling and H-NS DNA architectural properties

## SUPPLEMENTARY DATA

**Files in this Data Supplement:**

- SUPPLEMENTARY DATA
