# Supplementary material for: Single-molecule studies on the mechanical interplay between DNA supercoiling and H-NS DNA architectural properties
Source: Nucleic Acids Res. 2014 Jul 18;42(13):8369–78. doi: 10.1093/nar/gku566 (PMC4117784; doi:10.1093/nar/gku566)
Supplement: SUPPLEMENTARY DATA [file supp_gku566_nar-00811-m-2014-File008.pdf]

Single-molecule studies on the mechanical interplay between DNA supercoiling and H-NS DNA architectural properties
Lim, et al.

Supplementary Figures

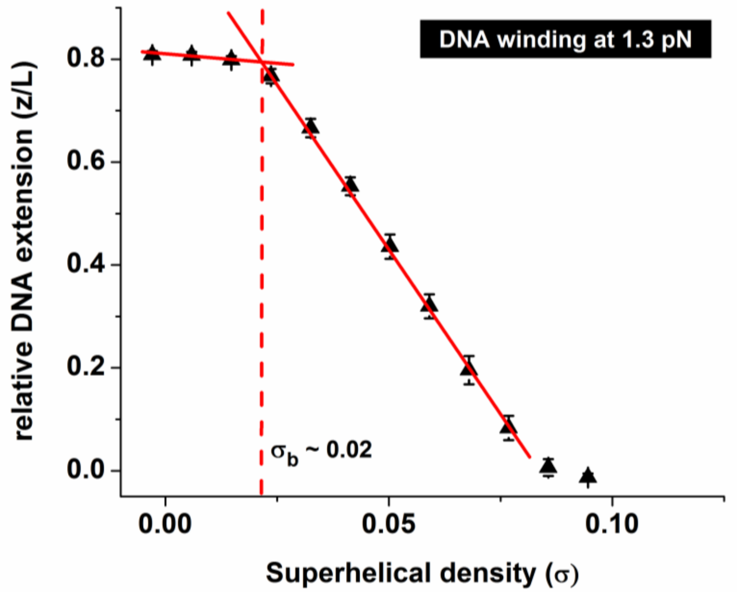

Figure S1. The DNA buckling transition measurement from DNA twist-extension curve. There are two regions of DNA extension vs. superhelical density (σ) that are approximately linear, which can be defined before and after DNA buckling transition (σ_b). At a given tension, at $\sigma < \sigma_b$, the DNA extension weakly depends on σ and is approximately linear with σ. At $\sigma > \sigma_b$, the DNA extension rapidly drops with σ as DNA twists are directly converted to writhes (plectonemes). At this region, the DNA extension decrease is linearly dependent on σ change. The transition point that separates the two linear regions defines the onset of DNA plectoneme formation and is thus defined as DNA buckling transition point. In experiments, this transition point can be determined by the interception between the two linear fits to the respective regions. Data shown in the figure were obtained at 1.30 pN during DNA winding. The two linear fits were optimized to obtain R2 of > 0.99, which interception determined the buckling transition point at σ_b = 0.021.

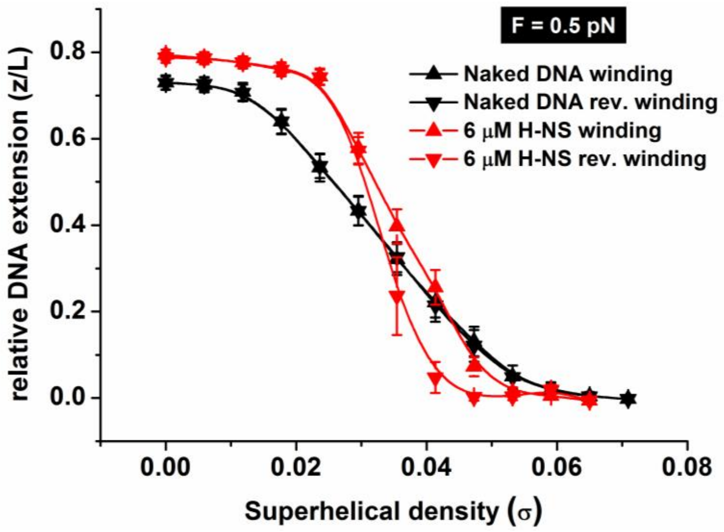

Figure S2. Saturated H-NS DNA-stiffening at 6,000 nM (6 μM) H-NS also restricts DNA conformational changes upon DNA winding (up-triangles) and reverse winding (down-triangles), which are indicated by a delayed buckling transition during winding and a hysteresis during reverse winding. The data shown was measured at 0.5 pN. The black symbols represent the naked DNA data while the red symbols represent data after introduction of 6 μM H-NS.

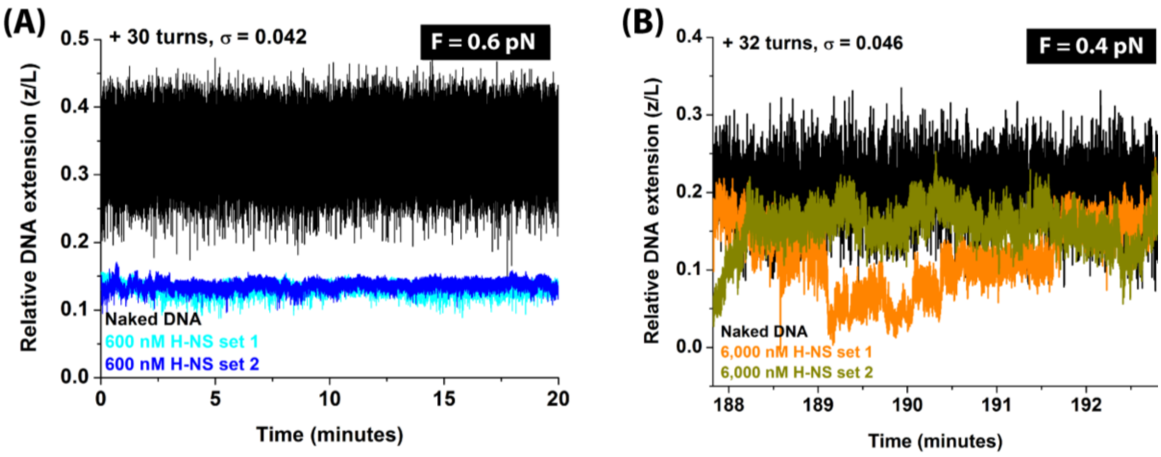

Figure S3. Time-trace of single DNA extension at constant low forces in the presence of H-NS DNA-stiffening mode at 600 nM (**A**) and 6,000 nM (6 μM) (**B**) concentrations after DNA bucking transition by DNA winding up to σ > 0.04. At each H-NS concentration, two independent data sets are shown. In all cases, by holding the DNA over 20 minutes, we did not observe any significant progressive DNA extension decrease, proving that the DNA extension drop during winding and the hysteresis during reverse winding are not caused by any progressive H-NS mediated DNA folding mechanisms.

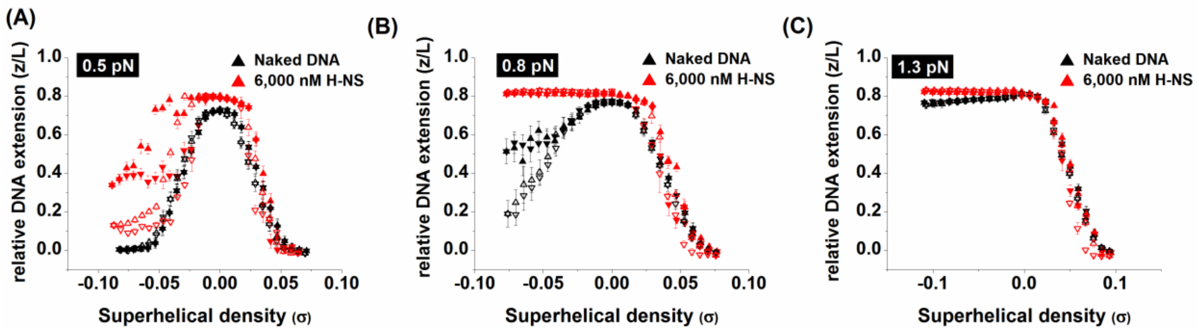

Figure S4. 6,000 nM (6 µM) H-NS in DNA-stiffening mode also promotes DNA melting during DNA unwinding. 2 independent data sets are plotted, indicated by solid symbols – set 1 and hollow symbols – set 2. Winding/unwinding curves are up-triangles while their reverse curves are down-triangles. (**A**) At 0.5 pN, H-NS DNA-stiffening caused partial DNA melting, while naked DNA formed DNA plectonemes. (**B**) At 0.8 pN, in the presence of H-NS DNA-stiffening, the DNA was completely melted, while the naked DNA was partially melted. (**C**) At 1.3 pN, both naked DNA and DNA in the presence of H-NS DNA-stiffening mode led to complete melting during DNA unwinding. This is similar to what was observed when a lower concentration of H-NS (600 nM) was used, as described in the main text.

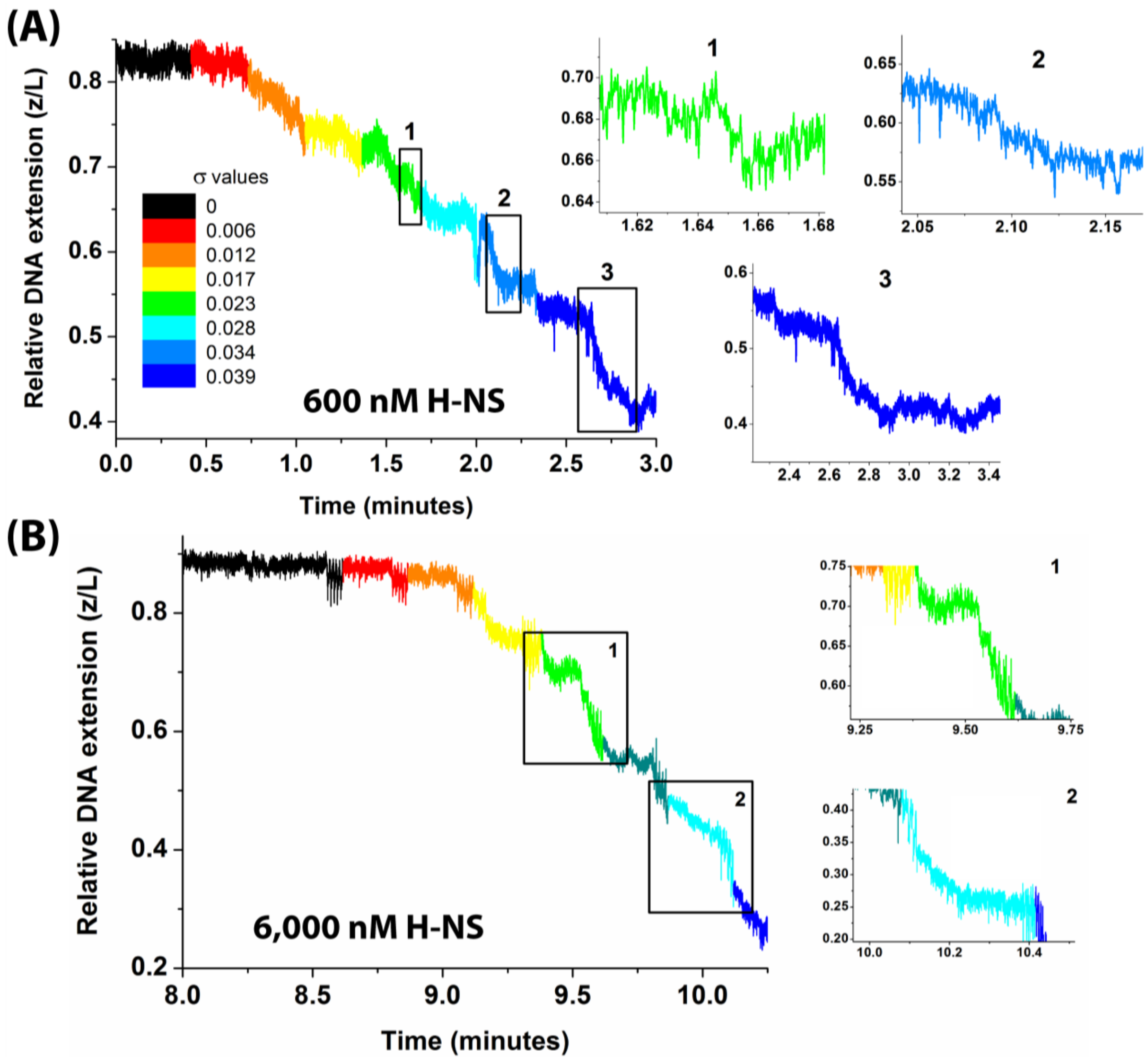

Figure S5. H-NS in DNA-bridging mode promoted DNA plectoneme formation. Progressive DNA folding indicated by progressive extension decrease was observed when DNA was held at constant DNA superhelical density values at 600 nM H-NS (**A**) and also 6,000 nM (6 µM) (**B**) at a DNA tension of 1.3-1.5 pN. Each winding caused a rapid extension reduction indicating more DNA was converted into plectonemes. The subsequent progressive DNA folding at the same value of σ suggests further zipping up of DNA by H-NS bridging mechanism. The insets are zoomed-in areas of progressive or step-wise DNA folding by H-NS.

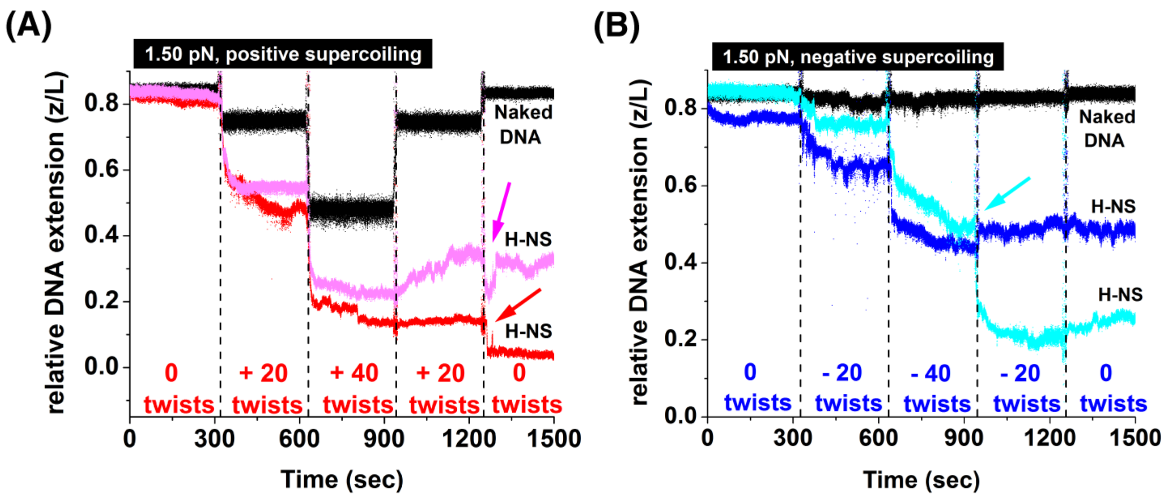

Figure S6. Time-course of DNA extension in the presence of (6,000 nM) 6 μM H-NS in DNA-bridging mode through a controlled DNA winding/reverse winding (**A**), positive supercoiling) and unwinding/ reverse unwinding (**B)**, negative supercoiling) cycles showed DNA plectoneme trapping similar to the case in 600 nM H-NS condition as described in the main text. DNA extension was not fully recovered upon reverse winding or reverse unwinding actions and instead resulted in further reduction of DNA extension (see arrows).
